# Limitations of a Cross-Sectional Correlation Study. Comment on Elabd et al. Prediction of Back Disability Using Clinical, Functional, and Biomechanical Variables in Adults with Chronic Nonspecific Low Back Pain. *J. Clin. Med.* 2024, *13*, 3980

**DOI:** 10.3390/jcm13195773

**Published:** 2024-09-27

**Authors:** Francisco M. Kovacs, Jesús Seco-Calvo

**Affiliations:** 1 Unidad de la Espalda Kovacs, Hospital Universitario HLA-Moncloa, Avda. Menéndez Pelayo Nº 67, 28009 Madrid, Spain; 2 Spanish Back Pain Research Network, Hospital Universitario HLA-Moncloa, Avda. Valladolid Nº 81, 28002 Madrid, Spain; jesus.seco@unileon.es; 3 Institute of Biomedicine (IBIOMED), University of León, 24071 León, Spain; 4 Visiting Researcher, Department of Physiology, University of the Basque Country, 48940 Leioa, Spain

We hereby comment on a study recently published by *J. Clin. Med.* [1]. We appreciate the effort undertaken by the authors to contribute to the existing literature exploring the correlation between disability and pain [2,3,4,5,6,7,8,9,10,11,12,13,14,15,16,17,18,19,20] in patients with low back pain (LBP). We would like to note the following:

1. As acknowledged by the authors, this study did not assess any psychosocial variables, such as fear avoidance, catastrophizing, anxiety, employment, litigation, or work-related factors. Previous studies, both with cross-sectional and longitudinal designs, have shown the influence of these variables on LBP-related disability and its evolution [2,3,4,5,6,7,8,9,10,11,12,13,14,15,16,17,18,19,20]. The magnitude of their influence and the specific psychosocial variables that are more relevant appear to vary across cultural settings [2,3,4,5,6,7,8,9,10,11,12,13,14,15,16,17,18,19,20]. However, no data are provided regarding their influence in the specific environment where this study took place. The lack of such data combined with the small sample size make it impossible to estimate to what extent variables that were not assessed in this study may account for its results, and whether the correlations found would have remained significant if these variables had been factored in.
2. As to be expected, especially in chronic LBP patients, the univariate analyses showed significant correlations between disability and pain, muscle endurance (as measured with the Sorensen test), and functional tests (i.e., sock test, pick-up test, roll-up test, fingertip-to-floor test, and lift test). However, in the multi-regression model, only the correlations between pain, disability, and muscle endurance remained significant. We think that this should have been emphasized when interpreting the results.
3. This was a correlation study with a cross-sectional design. Therefore, it can only establish correlations among different variables. The direction of the correlation can only be hypothesized, and the results from this study cannot prove any causality. For instance, the correlation between disability and low extensor muscle endurance in chronic patients may be interpreted as the latter being either the cause of the former or its consequence. We think that this should have been acknowledged and commented on in the Discussion section.
4. For the same reason, we think that the results from this study are not appropriate to support, challenge, or modify the current usual clinical practice. We do not question that multidisciplinary treatment is suitable for LBP, but we disagree that the results from this study serve to “emphasize” this need, identify “crucial” aspects that should be included in patient clinical assessment, or suggest any modifications to the currently recommended standard clinical practice. We think that further studies with the appropriate design are required for these purposes.
